# Best practices for portfolio optimization by quantum computing, experimented on real quantum devices

**DOI:** 10.1038/s41598-023-45392-w

**Published:** 2023-11-08

**Authors:** Giuseppe Buonaiuto, Francesco Gargiulo, Giuseppe De Pietro, Massimo Esposito, Marco Pota

**Affiliations:** Institute for High Performance Computing and Networking (ICAR), National Research Council of Italy (CNR), 80131 Naples, Italy

**Keywords:** Mathematics and computing, Computational science, Computer science, Information technology, Quantum physics

## Abstract

In finance, portfolio optimization aims at finding optimal investments maximizing a trade-off between return and risks, given some constraints. Classical formulations of this quadratic optimization problem have exact or heuristic solutions, but the complexity scales up as the market dimension increases. Recently, researchers are evaluating the possibility of facing the complexity scaling issue by employing quantum computing. In this paper, the problem is solved using the Variational Quantum Eigensolver (VQE), which in principle is very efficient. The main outcome of this work consists of the definition of the best hyperparameters to set, in order to perform Portfolio Optimization by VQE on real quantum computers. In particular, a quite general formulation of the constrained quadratic problem is considered, which is translated into Quadratic Unconstrained Binary Optimization by the binary encoding of variables and by including constraints in the objective function. This is converted into a set of quantum operators (Ising Hamiltonian), whose minimum eigenvalue is found by VQE and corresponds to the optimal solution. In this work, different hyperparameters of the procedure are analyzed, including different ansatzes and optimization methods by means of experiments on both simulators and real quantum computers. Experiments show that there is a strong dependence of solutions quality on the sufficiently sized quantum computer and correct hyperparameters, and with the best choices, the quantum algorithm run on real quantum devices reaches solutions very close to the exact one, with a strong convergence rate towards the classical solution, even without error-mitigation techniques. Moreover, results obtained on different real quantum devices, for a small-sized example, show the relation between the quality of the solution and the dimension of the quantum processor. Evidences allow concluding which are the best ways to solve real Portfolio Optimization problems by VQE on quantum devices, and confirm the possibility to solve them with higher efficiency, with respect to existing methods, as soon as the size of quantum hardware will be sufficiently high.

## Introduction

Portfolio Optimization (PO) is a fundamental financial task, with interesting applications in different scenarios, such as investment funds, pension schemes, and so on. Given a budget and/or a set of assets, it aims at finding optimal trades, within a market that can comprise a very high number of assets.

As formulated by Markowitz^1^, it can be expressed as a constrained quadratic optimization problem, where the objective function weighs different objectives including the maximal return and minimal risk (which constitute the quadratic term), subject to budget and/or other constraints. The optimization problem aims at finding optimal values of investments, which may be expressed as continuous variables (in terms of the fraction of budget to invest), but due to their discrete nature, are better represented as integers (in terms of the number of assets units to buy or sell) or binary variables (obtained by the binary encoding of integers).

If the general integer/binary version is regarded, combinatorial optimization is needed, which should find the right one among several tentative solutions that grow exponentially with the market dimension. Therefore, classical methods performing a brute-force approach to find an exact solution, such as the basic branch-and-bound method^2^, may present complexity issues. In practice, methods currently employed use heuristics to help the search of the branch-and-bound method^3^, or other methods such as Particle Swarms, Genetic Algorithms, and Simulated Annealing^4–6^. These approaches have some limitations but allow to obtain approximate solutions. In this work, the branch-and-bound method^3,7^ is used as a classical benchmark to find exact solutions and test the quality of the proposed approach.

To overcome these issues, this work considers that quadratic optimization problems are expected to be solved efficiently and with high accuracy on near-future quantum computers^8–12^. A detailed discussion about the complexity of different approaches is reported in the Methods section to motivate this alternative approach. In short, the possibilities offered by quantum effects might, in principle, promote quantum computers as valid trade-off solvers of NP-complete problems, giving improved performances in terms of approximation quality and computational time. Indeed, rather than looking for a provable global optimum, which may be unfeasible with hardware and performance limitations, quantum algorithms can find near-optimal solutions in acceptable computational time. This possibility is acquiring an interest in a growing number of fields, particularly relevant to optimization problems.

Optimization by Quantum Computing (QC) includes, on the one hand, quantum annealers^13^, designed to solve specific optimization problems by mapping them onto physical quantum Hamiltonian and have shown promising results when bench-marked with classically available algorithms. On the other hand, the real computational capabilities of gate-based quantum and hybrid algorithms are still to be explored. In particular, PO has been approached in a few works by quantum annealers^14,36^. At the same time, the solution of a simplified version of the problem by Variational Quantum Eigensolver (VQE) is available on the IBM Qiskit platform^15^. The performance of VQE, on classical simulators or on quantum devices, has been already successfully investigated in a number of works^16,17^. Other approaches exist, all based on gate quantum computers, that tackle the PO or other optimization problems. Quantum Approximate Optimization algorithms (QAOA) employ two types of quantum gates, a mixer Hamiltonian that facilitates parameterized mixing of quantum states, and a cost Hamiltonian that entails the optimization problem^18,19^. Refined versions of QAOA have been applied to PO, in simulated quantum environments^20^, showing promising results. The Grover mixer improves the performance of QAOA especially on constrained optimization^21^, as it is not sensitive to trotterization errors and solutions with equal values of the objective function are sampled with equal probability. Moreover, the quantum walk-based algorithm have been proven to be an effective way to improve the branch and bound algorithm^22^, giving an almost quadratic speed-up to the classical algorithm. Finally, as most of the interesting optimization problems present constraints that makes them hard to solve on quantum computers for their qubit overhead, it is worth mentioning here the parity mapping approach^23^, which introduces an alternative way to the standard spin Hamiltonian encoding approach making use only of parity variables, thus reducing the complexity of the problem.

Until nowadays, the size of quantum devices is limited, and the computation is not fault-tolerant, i.e., an efficient quantum error correction is not available yet. But very recently, the availability of Noisy Intermediate Scale Quantum (NISQ) devices allows testing the performances of both quantum and hybrid algorithms to explore new computational paradigms that find various applications in fields such as chemistry, biology^24–26^, and artificial intelligence^27^. Furthermore, error mitigation techniques are available, which allow to estimate and hence reduce the effect of the noise on the quantum algorithms^28^. However, a detailed study of the performances of various NISQ devices for PO is still missing.

In this work, PO is approached by QC, specifically making use of the VQE algorithm to find an optimal or sub-optimal solution to the problem. Although the VQE methodology has been extensively used in various scenarios, a complete experimental analysis of this technique, including the encoding strategy, on a significant subset of the actual state-of-the-art quantum machines is still missing. With respect to the state-of-the-art approaches for PO with QC, VQE is chosen instead of quantum annealers for its generality. The particular choice of focusing on VQE only in the present work is dictated by the need of benchmarking the performances of a robust and yet general algorithm on real quantum devices pointing at the change in the quality of the results depending of hardware structural factors (the specification of the quantum computer in use) and algorithmic factors (variational ansatz and optimizers in particular). Therefore, the existing simplified version available on Qiskit^15^ is generalized into the general optimization problem. Moreover, an experimental investigation is performed using real IBM quantum computers. This is the first work that cross-relates different aspects of the VQE quantum algorithm with those of the real quantum hardware, to obtain the best performances for the specific PO problem. In particular, this work presents solutions to the problem obtained on different quantum computers and with different hyperparameters settings, to find the best practices to perform PO by VQE on real quantum devices.

More in detail, a sample of a limited size of real financial data is employed since, albeit the scale of the system considered does not match realistic requirements, it allows exploring the efficiency of the QC approach. These data are used to construct the objective as a trade-off between the expected return and variance, weighted by a risk aversion coefficient. Then, the general constrained integer quadratic formulation is transformed into a Quadratic Unconstrained Binary Optimization (QUBO) problem through the binary encoding of variables, designed to reduce the required number of qubits, and by a penalty coefficient, which weights constraints satisfaction with respect to objectives, to include constraints into the objective function^36^. This is converted into a set of quantum operators (Ising Hamiltonian), whose minimum eigenvalue corresponds to the optimal solution. An approximation of this solution is found by VQE, a hybrid algorithm involving the choices of a parametric tentative quantum state (ansatz), and a classical optimization algorithm.

Therefore, this paper aims to find the best hyperparameters settings to perform PO by VQE, i.e., appropriate ansatz and optimizer are found so that the effect of noise is minimized. At the same time, the convergence rate is maximized, and a suitable penalty coefficient is found based on its effect on the convergence toward the correct solution. Finally, the optimal solutions are compared among those obtained on simulators and on real quantum computers of different sizes and architectures and with the benchmark solution.

The paper is structured as follows: firstly, the materials and methods are presented, including the dataset description, the formulation of the PO problem, its translation into a QUBO problem, then into a quantum Hamiltonian, the generalities of the VQE method, and details of the IBM NISQ devices. In the following section, results are shown and discussed. Finally, conclusions and future perspectives are outlined.

## Materials and methods

### Dataset

The data are collected from Yahoo!@finance^29^ using *yfinance*^30^, an open-source tool that uses Yahoo’s publicly available APIs. This tool, according to its creator, is intended for research and educational purposes.

To explore the efficiency of the proposed approach, small-sized examples are considered by extracting at most $N=4$ different assets: $\text {Apple}$, $\text {IBM}$, $\text {Netflix}$ and $\text {Tesla}$. These are representative global assets with interesting dynamics influenced by financial and social events. For each asset *i*, with $1\le i \le N$, the temporal range between 2011/12/23 and 2022/10/21 is considered. For each day *t* in this range ($0\le t \le T$), the performance of an asset is well represented by its closing price $p^t_i$. A sub-interval of dates considered is shown in Table 1. Additional experiments, performed on different dataset and falling within the same time interval considered here, are available in the supplementary information.

**Table 1:** Closing prices of four assets, *Apple*, *IBM*, *Netflix* and *Tesla*, for a sub-interval of the whole time period, extracted from Yahoo!@finance using *yfinance* Python package, and considered for experiments in this work.

Date	AAPL	IBM	NFLX	TSLA
2016-12-23	27.219765	119.262428	125.589996	14.222667
2016-12-27	27.392632	119.570061	128.350006	14.635333
2016-12-28	27.275822	118.890434	125.889999	14.649333
2016-12-29	27.268820	119.183701	125.330002	14.312000
2016-12-30	27.056236	118.747368	123.800003	14.246000
2017-01-03	27.133329	119.605820	127.489998	14.466000
2017-01-04	27.102961	121.086693	129.410004	15.132667
2017-01-05	27.240784	120.686058	131.809998	15.116667
2017-01-06	27.544472	121.279861	131.070007	15.267333
2017-01-09	27.796768	119.934898	130.949997	15.418667
2017-01-10	27.824797	118.411110	129.889999	15.324667
2017-01-11	27.974308	120.006439	130.500000	15.315333

The first information extracted from this data set consists in the list *P* of current prices $P_i$ of the considered assets.

1 $$\begin{aligned} P_{i}=p^T_i. \end{aligned}$$

Moreover, for each asset, the return $r^t_i$ between the days $t-1$ and *t* can be calculated:

2 $$\begin{aligned} r^t_i=\frac{p^t_i-p^{t-1}_i}{p^{t-1}_i} \end{aligned}$$

These returns, calculated for days when the initial and the end prices are known, cannot be used for inference. Instead, it is convenient to define the expected return of an asset as an educated guess of its future performance. Assuming a normal distribution of the returns, the average of their values at each time *t* on the set of historical observations is a good estimator of the expected return. Therefore, given the entire historical data set, the expected return of each asset $\mu _i$ is calculated by:

3 $$\begin{aligned} \mu _i=E[r_i]=\frac{1}{T}\sum _{t=1}^{T}r^t_i. \end{aligned}$$

Following the same principle, the variance of each asset return and the covariance between returns of different assets over the historical series can be calculated as follows:

4 $$\begin{aligned}&\sigma ^{2}_{i}=E[(r_{i}-\mu _{i})^2]=\frac{1}{T-1}\sum _{t=1}^{T}(r^{t}_{i}-\mu _{i})^{2}, \\&\sigma _{ij}=E[(r_{i}-\mu _{i})(r_{j}-\mu _{j})]=\frac{1}{T-1}\sum _{t=1}^{T}((r^{t}_{i}-\mu _{i})(r^{t}_{j}-\mu _{j})) \nonumber . \end{aligned}$$

### Portfolio optimization

The traditional theory of PO was initially formulated by Markowitz^1^. There are multiple possible formulations of PO, all embodying different degrees of approximation of the real-life problem. This work deals with Multi-Objective Portfolio optimization: this approach tries to simultaneously maximize the return and minimize the risk while investing the available budget. Even if other formulations include more objectives, the aim is still the solution of a constrained quadratic optimization problem; therefore, the formulation considered here is general enough to test the performances of the proposed approach.

A portfolio is defined as the set of investments $x_{i}$ (measured as a fraction of the budget or number of asset units) allocated for each *i*th asset of the market. Therefore, the portfolio consists of a vector of real or integer numbers with dimensions equal to the number of assets considered. An optimal strategy for portfolio allocations aims to achieve the maximum portfolio return $\mu ^{\text {T}} x$ while minimizing risk, defined as the portfolio variance $x^{\text {T}}\Sigma x$ (whose square root is the portfolio volatility), where $\mu$ is the vector of mean asset returns for each asset *i* calculated by (3), $\Sigma$ is the covariance matrix calculated by (4), and *x* is the vector of investments measured as fractions of budget. Hence, the task of finding the optimal portfolio aims at finding the *x* vector that maximizes the following objective function:

5 $$\begin{aligned} {{\mathscr {L}}}(x): \mu ^{\text {T}} x - qx^{\text {T}}\Sigma x, \end{aligned}$$

where the risk aversion parameter *q* expresses the propensity to risk of the investor (a trade-off weight between the risk and the return).

In a realistic scenario, the available budget *B* is fixed. Therefore, the constraint that the sum of $x_i$ equals 1 must hold. Moreover, if only buying is allowed, each $x_i\ge 0$, this constraint does not hold if either buying or selling is possible. As a consequence, in the general case, the problem can be stated as follows:

6 $$\begin{aligned}&\underset{x}{\max }{\mathscr {L}}(x): \underset{x}{\max }(\mu ^{\text {T}} x - qx^{\text {T}}\Sigma x),\\&\text {s.t.} \quad \sum ^{N}_{i=1}x_i=1 \nonumber \end{aligned}$$

However, if *x* is a possible solution to the problem with continuous variables, each product $x_iB$ must be an integer multiple of the corresponding price $P_i$ calculated by (1) since an integer number of units of each asset can be exchanged. Therefore, only a subset of the possible solutions corresponding to integer units is acceptable, and the problem is better stated as follows:

7 $$ \begin{gathered}   \mathop {\max }\limits_{n} {\mathcal{L}}(n):\mathop {\max }\limits_{n} (\mu ^{{\prime {\text{T}}}} n - qn^{{\text{T}}} \Sigma ^{\prime } n), \hfill \\   {\text{s}}{\text{.t}}{\text{.}}\quad P^{{\prime {\text{T}}}} n = 1 \hfill \\  \end{gathered}  $$

where *n* is the vector of $n_i$ integer units of each asset, while $P'=P/B$, $\mu '=P'\circ \mu$ and $\Sigma '=(P'\circ \Sigma )^{\text {T}}\circ P'$ are appropriate transformations of $\mu$ and $\Sigma$. The latter formulation (7) is an integer constrained quadratic optimization problem.

Possible solutions to the problem (6) are those satisfying the constraint. Among them, some correspond to possible solutions to problem (7). The collection of possible solutions corresponding to portfolios with maximum return for any risk is called “Markowitz efficient frontier”. The solution of the constrained quadratic optimization problem lies on the efficient frontier, and the distance from minimum risk depends on *q*.

### Complexity

The general problem, if regarded in terms of continuous variables, can be solved exactly by Lagrange multipliers in case of equality constraints, or by Karush–Kuhn–Tucker conditions, which generalize the method of Lagrange multipliers to include inequality constraint^31^, as the covariance matrix is positive semi-definite^32^. Optimizing a quadratic function subject to linear constraints leads to a linear system of equations, solvable by Cholesky decomposition^33^ of the symmetrical covariance matrix. The exact solution involves the computation of the inverse of an $N \times N$ matrix, where *N* is the number of assets, thus requiring about $O(N^3)$ floating-point operations^34^.

As long as integer or binary variables are considered, the problem turns into combinatorial optimization. The computational complexity is known to be high since the optimization problem is NP-hard^35,36^, while the decision version is NP-complete^37^. Indeed, a search approach should find the optimal one among possible solutions whose number increases exponentially with the number of assets (e.g., for *b* binary variables, $2^b$ possible solutions, while for *N* integer variables ranging from 0 to $n_{max}$, ${\left( n_{max}+1\right) }^N$ possible solutions).

In practice, various methods are currently employed, either based on geometric assumptions, such as the branch-and-bound method^2,3^, or rather heuristic algorithms^4–6^, such as Particle Swarms, Genetic Algorithms, and Simulated Annealing. These have some limitations but allow to obtain approximate solutions. However, in all cases, the exact or approximate solution is feasible only for a few hundreds of assets on current classical computers.

Using quantum mechanical effects, like interference and entanglement, quantum computers can perform computational operations within the Bounded-error Quantum Polynomial (BQP) class of complexity, which is the quantum analogue of the Bounded-error polynomial probabilistic (BPP) class. Even if there is no NP problem for which there is a provable quantum/classical separation, it is widely believed that BQP $\not \subset$ BPP, hence when considering time complexity, quantum computers are more powerful than classical computers. More generally, it is conjectured that P is a subset of BQP. Therefore, while all problems that can be efficiently solved classically, are efficiently solvable by quantum computers as well, some problems exist that are considered intractable, until nowadays, by classical computers in polynomial space, and can they be solved with quantum machines. These facts are still matter of investigation but there are good reasons to believe that there are problems solvable by QC more efficiently than classical computers, thus QC will have a disruptive potential over some hard problems^38^, among which constrained quadratic optimization problems, including PO.

### Classical solution

The branch-and-bound method^3,7^ is used in this work as a classical benchmark to compare the results of the proposed approach. It is based on the Lagrangian dual relaxation and continuous relaxation for discrete multi-factor portfolio selection model, which leads to an integer quadratic programming problem. The separable structure of the model is investigated by using Lagrangian relaxation and dual search. This algorithm is capable of solving portfolio problems with up to 120 assets.

Specifically, the library CPLEX freely available on Python provides a robust implementation of the aforementioned classical solving scheme.

### Quantum formulation

As formulated in Eq. (7), the PO problem lies within the class of quadratic optimization problems. To be quantum-native, it has to be converted into a Quadratic Unconstrained Binary Optimization (QUBO) problem, i.e., the target vector to be found has to be expressed as a vector of zeros and ones, and constraints have to be avoided.

Therefore, the binary conversion matrix *C* is constructed with a number of binarizing elements $d_i$ for each asset *i* depending on the price $P_i$. Hence

8 $$\begin{aligned} n^{max}_{i}=Int\left( \frac{B}{P_i}\right) , \end{aligned}$$

where the operation *Int* stands for the integer part, and

9 $$\begin{aligned} d_i=Int\left( \log _{2}{n^{max}_i}\right) , \end{aligned}$$

such that

10 $$\begin{aligned} n_{i}=\sum _{j=0}^{d_i}2^{j}b_{i,j}. \end{aligned}$$

In this way, the overall dimension of the binarized target vector, $b=\left[ b_{1,0},\dots ,b_{1,d_1},\dots ,b_{N,0},\dots ,b_{N,d_N}\right]$, is $\text {dim}(b) =\sum _{i=1}^{N}\left( d_i+1\right)$, which is lower than that used in implementation available in Qiskit^15^. Conveniently, the encoding matrix *C* is defined as follows:

11 $$\begin{aligned} C= \begin{pmatrix} 2^{0} &{} \dots &{} 2^{d_1} &{} 0 &{} \dots &{} 0 &{} \dots &{} 0 &{} \dots &{} 0 \\ 0 &{} \dots &{} 0 &{} 2^{0} &{} \dots &{} 2^{d_2} &{} \dots &{} 0 &{} \dots &{} 0 \\ \vdots &{} \ddots &{} \vdots &{}\vdots &{} \ddots &{}\vdots &{} \ddots &{}\vdots &{}\ddots &{}\vdots \\ 0 &{} \dots &{} 0 &{} 0 &{} \dots &{} 0 &{} \dots &{} 2^{0} &{} \dots &{} 2^{d_N} \end{pmatrix}, \end{aligned}$$

and thus, the conversion can be written in short notation as $n = Cb$. It is possible to redefine the problem (7), in terms of the binary vector *b*, applying the encoding matrix by $\mu ''=C^{\text {T}}\mu '$, $\Sigma ''=C^{\text {T}}\Sigma 'C$ and $P''=C^{\text {T}}P'$:

12 $$ \begin{gathered}   \mathop {\max }\limits_{b} {\mathcal{L}}(b):\mathop {\max }\limits_{b} \left( {\mu ^{{\prime \prime {\text{T}}}} b - qb^{{\text{T}}} \Sigma ^{{\prime \prime }} b} \right), \hfill \\   {\text{s}}{\text{.t}}{\text{.}}\quad P^{{\prime \prime {\text{T}}}} b = 1 \hfill \\   \quad \quad b_{i}  \in \{ 0,1\} \quad \forall i \in \left[ {1, \ldots ,dim(b)} \right]. \hfill \\  \end{gathered}  $$

The problem (12) falls into the wide set of binary quadratic optimization problems, with a constraint, given by the total budget. In this form, the problem cannot be cast directly into a suitable set of quantum operators that run on quantum hardware: the constraint, in particular, is troublesome, as it poses a hard limitation on the sector of Hilbert space that needs to be explored by the algorithm, to find a solution. It is thus necessary to convert the problem into a QUBO (Quadratic Unconstrained Binary Optimization) by transforming the constraint into a penalty term in the objective function. Each kind of constraint can be converted into a specific penalty term^39^, and the one considered in (12), which is equality, linear in the target variable, maps into $\lambda (P''^{\text {T}} b-1)^{2}$, such that (12) can be written in terms of the following QUBO problem:

13 $$ \mathop {\max }\limits_{b} {\mathcal{L}}(b):\mathop {\max }\limits_{b} \left( {\mu ^{{\prime \prime {\text{T}}}} b - qb^{{\text{T}}} \Sigma ^{{\prime \prime }} b - \lambda (P^{{\prime \prime {\text{T}}}} b - 1)^{2} } \right). $$

The penalty coefficient $\lambda$ is a key hyperparameter to state the problem as the QUBO of the objective function (13).

There is a strong connection, technically an isomorphism, between the QUBO and the Ising Hamiltonian^40^: Ising Hamiltonian was originally constructed to understand the microscopic behavior of magnetic materials, particularly to grasp the condition that leads to a phase transition. However, its relative simplicity and natural mapping into QUBO have made the Ising model a fundamental benchmark well beyond the field of quantum physics. To convert (13) into an Ising, it is convenient to expand it in its components:

14 $$ {\mathcal{L}}(b):\sum\limits_{i} {\mu _{i}^{\prime } b_{i} }  - q\sum\limits_{{i,j}} {\Sigma _{{i,j}}^{\prime } } b_{i} b_{j}  - \lambda \left( {\sum\limits_{i} {P_{i}^{\prime } b_{i}  - 1} } \right)^{2} , $$

where $\mu ''_{i}, \Sigma ''_{i,j}, P''_{i}$, are the components of the transformed return, covariance, and price, respectively, and $i,j\in \left[ 1,dim(b)\right]$. Since the Ising represents spin variables $s_{i}$, which have values $\{-1,1\}$, the transformation $b_{i}\rightarrow \frac{1+s_{i}}{2}$ is applied and coefficients are re-arranged, to obtain the Ising objective function to minimize:

15 $$\begin{aligned}&\underset{s}{\min }{\mathscr {L}}(s): \underset{s}{\min }\left( \sum _{i}h_{i}s_{i}+ \sum _{i,j} J_{i,j}s_{i}s_{j}+\lambda (\sum _{i}\pi _{i}s_{i}-\beta )^{2}\right) ,\\&\text {s.t.} \quad s_{i,j}\in \{-1,1\} \quad \forall i \nonumber , \end{aligned}$$

with $J_{i,j}$ being the coupling term between two spin variables. It is now straightforward to obtain the corresponding quantum Hamiltonian, whose eigenvector corresponding to the minimum eigenvalue corresponds to the solution: in fact, the eigenvalues of the Pauli operators *Z* are $\pm 1$. Thus they are suitable for describing the classical spin variables $s_{i}$. Furthermore, the two-body interaction term can be modeled with the tensor product between two Pauli operators, i.e., $Z_{i}\otimes Z_{j}$. The quantum Ising Hamiltonian reads:

16 $$\begin{aligned} H= \sum _{i}h_{i}Z_{i} + \sum _{i,j} J_{i,j}Z_{i}\otimes Z_{j}+\lambda (\sum _{i}\pi _{i}Z_{i}-\beta )^{2}. \end{aligned}$$

With the procedure described above, the integer quadratic optimization problem of a portfolio allocation with budget constraints is expressed first as a binary problem via the binary encoding, then it is translated into a QUBO, transforming the constraints into a penalty term by the chosen penalty coefficient, and finally into a quantum Hamiltonian written in term of Pauli gates. Hence, the PO problem (7) is now formulated as the search of the ground state, i.e., the minimum energy eigenstate, of the Hamiltonian (16). Therefore, it is possible to use the VQE, employing real quantum hardware, and iteratively approximate such a state, as described in the following section, which corresponds to the optimal portfolio.

### Variational Quantum Eigensolver

The VQE is a hybrid quantum-classical algorithm^41^, which is based on the variational principle: it consists in the estimation of the upper bound of the lowest possible eigenvalue of a given observable with respect to a parameterized wave-function (ansatz). Specifically, given a Hamiltonian *H* representing the observable, and a parameterized wave-function ${|{\psi (\theta )}\rangle }$, the ground state $E_{0}$ is the minimum energy eigenstate associated s

17 $$\begin{aligned} E_{0}\le \frac{{\langle {\psi (\theta }|}H{|{\psi (\theta )}\rangle }}{{\langle {\psi (\theta )|\psi (\theta )}\rangle }}, \quad \forall \quad \theta . \end{aligned}$$

Hence, the task of the VQE is finding the optimal set of parameters, such that the energy associated with the state is nearly indistinguishable from its ground state, i.e., finding the set of parameters $\theta$, corresponding to energy $E_{min}$, for which $|E_{min}-E_{0}|<\epsilon$, being $\epsilon$ an arbitrarly small constant. This problem can be formulated on a quantum computer as a series of parameterized quantum gates, which are applied on the initial state to realize a structured ansatz for the Hamiltonian problem. Conventionally, the initial state is set to be the vacuum state, i.e., for *Q* qubit system ${|{0}\rangle }^{\otimes Q}={|{\textbf{0}}\rangle }$, where $\otimes$ stands for the tensor product between each state describing the single qubit system. Thus, on a quantum device, the problem of maximizing the objective function (17) can be expressed as:

18 $$\begin{aligned} E_{\text {min}}=\underset{\theta }{\min }{\langle {\textbf{0}}|}U^{\dagger }(\theta )HU(\theta ){|{\textbf{0}}\rangle }. \end{aligned}$$

where $U(\theta )$ is the parametrized unitary operator that gives the ansatz wave-function when applied on the initial state, $E_{min}$ is the energy associated with the parametrized ansatz. The Hamiltonian *H*, defined for the specific problem, and in this case corresponding to (16), can be written in a specific operator basis that makes it naturally measurable on a quantum computer: this choice depends on the architecture considered. In this work, given the extensive use of the IBM quantum experience^42^, it is convenient to map the Hamiltonian into spin operators’ base. This base is formed by the tensor product of Pauli strings: $P_{l}\in \{I,X,Y,Z\}^{\otimes N}$. In this base the Hamiltonian can always be written in the general form, $H=\sum _{l}^{D}c_{l}P_{l}$, where *D* is the number of Pauli strings that define the Hamiltonian and $c_{l}$ is a suitable set of weights. It follows that the VQE in Eq. (18) can be written as:

19 $$\begin{aligned} E_{\text {min}}=\underset{\theta }{\min }\sum _{l}^{D}c_{l}{\langle {\textbf{0}}|}U^{\dagger }(\theta )P_{l} U(\theta ){|{\textbf{0}}\rangle }. \end{aligned}$$

Each term in Eq. (19) corresponds to the expectation value of the string $P_{l}$ and is computed on quantum hardware (or a simulator). The summation and the optimization of the parameters are computed on a classical computer, choosing an ad-hoc optimizer. The eigenvector corresponding to the ground state corresponds to the solution of the problem(13), thus to the optimal portfolio.

**Figure 1:**
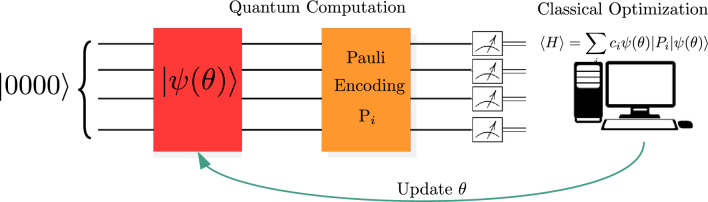
Schematic of the VQE algorithm. The ansatz wave-function ${|{\psi (\theta )}\rangle })$ is initialized with random parameters and encoded in a given set of quantum gates. The PO problem is translated into an Ising Hamiltonian and encoded into a set of Pauli gates. The collection of output measurement allows the reconstruction of the expectation value of the Hamiltonian *H*, which is the energy that needs to be minimized. A classical optimization algorithm provides an update rule for the parameters of the wave-function, which ideally moves iteratively towards the ground state of the problem, thus providing an estimation of the corresponding eigenstate. This corresponds to the solution of the original PO problem.

In light of what is stated above, the complete VQE estimation process can be decomposed in a series of steps, as depicted in Fig. 1. First, it is necessary to prepare a trial wave-function (ansatz) on which the expectation value needs to be evaluated and realized via a parameterized quantum circuit. Then, it is necessary to define the Hamiltonian (16), whose ground state is the solution to the problem to be addressed, and convert it into the Pauli basis so that the observable can be measured on the quantum computer. Finally, the parameters are trained using a classical optimizer. This hybrid system ideally converges to a form that produces a state compatible with the ground state of the Hamiltonian.

This procedure includes two hyperparameters that have to be settled, i.e., the type of ansatz and the optimizer. When defining the ansatz, two main features have to be taken into account: its expressivity, i.e., the set of states that can be spanned by the ansatz itself, and the trainability, i.e., the ability of the ansatz to be optimized efficiently with available techniques. It is worth pointing out the problem of the barren plateau^43^, related to the possibility of vanishing gradients when the cost function gradients converge to zero exponentially, as a function of the specific characteristic of the problem to be solved. The barren plateau depends on the number of qubits, the high expressivity of the ansatz wave-function, the degree of entanglement, and the quantum noise^44^. There are several methods to avoid or mitigate the effect of the barren plateau, especially in the context of VQE, most of which consist in finding a trade-off between the expressivity of the ansatz and its trainability and reducing the effective size of the Hilbert space of the problem formulation^45^.

The following ansatzes are available in Qiskit and are analyzed in this work: *Two Local* ansatz, where qubits are coupled in pairs, the *Real Amplitude* ansatz, which assumes real-valued amplitude for each base element of the wave-function, and the *Pauli Two* ansatz, used mainly in quantum machine learning for the mitigation of barren plateu^46^. Although other ansatzes are provided in Qiskit, they are generally unsuitable for a PO problem. For instance, the *Excitation preserving* ansatz preserves the ratio between basis vector components, hence does not allow, in principle, any weight imbalance in the output distribution while moving towards the solution of the problem.

For all the ansatzes considered, the convergence of four different possible assumptions on the entanglement structure of the wave-function is checked, namely the *full* entanglement, the *linear* entanglement, the *circular* and the *pairwise* entanglement. The former modifies the ansatz such that any qubit is entangled with all the others pairwisely. In the linear case, the entanglement is built between consecutive pairs of qubits. The circular case is equivalent to the linear entanglement but with an additional entanglement layer connecting the first and the last qubit before the linear sector. Finally, in the pairwise entanglement construction, in one layer, the *i*th qubit is entangled with qubit $i+1$ for all even *i*, and in a second layer, qubit *i* is entangled with qubit $i+1$, for odd values of *i*.

Once the ansatz is defined, its parameters must be optimized classically until convergence is reached. The choice of the optimizer is crucial because it impacts the number of measurements that are necessary to complete the optimization cycle since, when properly chosen, it can mitigate the barren plateau problem and minimize the number of iterations required to reach convergence. In this work, dealing with the PO problem, different optimizers are tested to select which one fulfills its task faster, among those available on Qiskit, i.e., *Cobyla*, *SPSA*, and *NFT*^47^.

### NISQ devices

The experimental results presented in this work are obtained on real quantum hardware, specifically using the platforms provided by IBM superconducting quantum computers. These quantum machines belong to the class of NISQ devices, which stands for Noisy Intermediate Scale Quantum devices, i.e., a class of hardware with a limited number of qubits and where noise is not suppressed. Noise, in quantum computers, comes from various sources: decoherence, gate fidelities, and measurement calibration. Decoherence is the process that most quantum mechanical systems undergo when interacting with an external environment^48^. It causes the loss of virtually all the quantum properties of the qubits, which then collapse into classical bits. Gate fidelities measure the ability to implement the desired quantum gates physically: in the IBM superconducting qubits hardware, these are constructed via pulses, which are shaped and designed to control the superconductors. Given the limited ability to strictly control these pulses, a perfect gate implementation is highly non-trivial and subject to imperfections. Last, measurement errors are caused by the limits of the measurement apparatus, improper calibration, and imperfect readout techniques. Hence, NISQ devices do not always provide reliable results due to the lack of fault tolerance. However, they provide a good benchmark for testing the possibilities of quantum computing. Furthermore, ongoing research is on the possibility of using NISQ in practical applications, such as machine learning and optimization problems.

In this work, both simulators and real quantum computers are used. Even though error mitigation techniques^49^ can be applied, the main goal of this paper is to test the performances of the quantum computers on a QUBO problem, such as PO, without error mitigation, with the binary encoding strategies and the budget constraints as described in the previous sections. Therefore, in all computations, there is no error mitigation, aiming to build an indirect but comprehensive analysis of the hardware limitations and to improve the quality of the results offered by a proper selection of the hyperparameters. This will provide a solid benchmark for the following experimental stages, which will be enabled in the coming years by large and nearly fault-tolerant quantum computers.

Hence, the experiments run on simulators (without noise) are also executed by adding noise mimicking real hardware: this operation can be readily implemented on Qiskit by inserting a noise model containing the decoherence parameters and the gate error rate from real quantum hardware.

Moreover, experiments are run on IBM NISQ devices with up to 25 qubits. Specifically, a substantial subset of the available quantum computers in the IBM quantum experience was employed: IBM Guadalupe, Toronto, Geneva, Cairo, Auckland, Montreal, Mumbai, Kolkata, and Hanoi. These machines have either 16 or 27 qubits, but they have different quantum volumes (QV) and Circuit Layer Operations Per Second (CLOPS). QV and CLOPS are useful metrics to define the performances of a quantum computation pipeline^50^. Generally, a bigger QV means that the hardware can sustain deeper circuits with a relatively small price on the performance. At the same time, the CLOPS quantifies the number of operations that can be handled by the hardware per unit of time. Hence, altogether, they qualify the quality and speed of quantum computation.

## Results and discussion

In this section, PO results obtained with different hyperparameters and on different simulated and real quantum devices are presented and discussed in terms of the algorithm’s convergence and the quality of the optimal solution found.

### Experimental settings

The experiments involve all the assets described in the Dataset section. Prices are calculated from data by Eq. (1), mean returns by (3), and covariance matrix by (4). The total expendable budget *B* is set to be commensurate with the number of assets considered, i.e., $B=2000$ for all experiments. All the experiments are executed by considering the risk aversion parameter $q=0.5$, representing a mid-way compromise between the risk and the return.

The proposed approach is implemented on the Qiskit^51^ software development kit. A set of 12 qubits was required to encode the spin variables of Eq. (15), to encode the binarized number of investments. Initial ansatz parameters were set randomly between $-\pi$ and $\pi$. For each instance of the VQE, the average value over a set of 2000 runs is considered. Results with varying hyperparameters are obtained by quantum simulators, with and without simulated noise. The best set of hyperparameters is used in the experiments on different real quantum computers.

### Study of hyperparameters

The proposed approach involves the setting of methods and constants related to the optimization algorithm and the QUBO formalization of the problem. In this section, results obtained with different settings of hyperparameters are presented to find the best choices for PO. Further experiments, on different data, proving the robustness of the result here presented can be found in the Supplementary Information.

**Figure 2:**
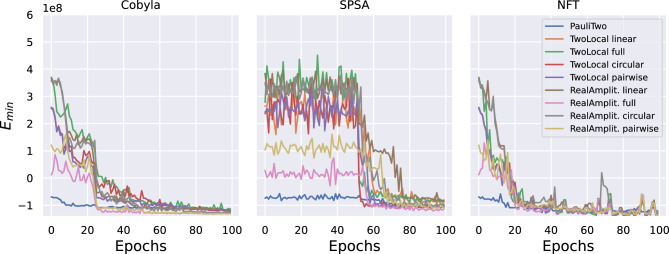
Noiseless experiments performed on IBM QASM simulator, supposing a fault-tolerant quantum machine, with no quantum noise influencing the quality of the results. Convergence of the solutions towards the optimal one during training epochs, evaluated with different optimizers and different ansatzes. For all these experiments, a penalty term $\lambda =10$ was used.

**Figure 3:**
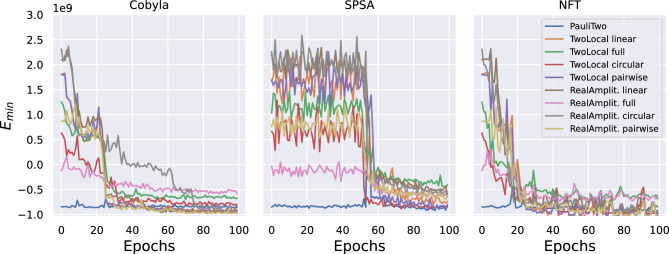
Noisy experiments, performed on IBM QASM simulator, by importing IBM Cairo quantum computer noise model. Convergence of the solutions towards the optimal one during training epochs, evaluated with different optimizers and ansatzes. For all these experiments, a penalty term $\lambda =10$ was used.

Firstly, the hybrid VQE algorithm entails the choice of a type of ansatz to initialize the qubit wave-function, and the choice of a classical optimizer to tune the ansatz parameters toward the solution, as detailed in the VQE Section. In Figs. 2 and 3, the convergence of experiments performed by using different ansatzes and optimizers are reported. Both figures show the convergence rate, during epochs, towards the minimum energy $E_{min}$, which approximates the ground energy of the Hamiltonian and corresponds to the quality of the solution of the PO problem.

In particular, Fig. 2 reports results obtained on a noiseless quantum simulator, while Fig. 3 shows more noisy experiments on a simulated noisy quantum computer. Specifically, the first set of experiments is performed with the QASM quantum simulator provided by IBM^52^, while the second set is done on the same simulator by importing the noise model from the specifications of the IBM Cairo quantum computer. In both cases, the experiments are performed over a set of nine ansatzes and three possible optimizers, all provided by Qiskit^47^, as detailed in the Methods section.

Regarding the comparisons among classical optimizers, both figures allow some considerations. First, both the Cobyla and the NFT optimizers foster a rapid convergence towards low values of the energy for every ansatz, while SPSA presents a delayed behavior. On the other hand, the NFT^53^ optimizer, contrarily to the others, experiences relatively unstable behavior, with highly oscillating trajectories for each ansatz. Moreover, all optimizers are relatively robust against statistical noise, but a more oscillating behavior is obtained in noisy simulations using SPSA or NFT optimizers. This preliminary analysis suggests that the Cobyla optimizer is the most stable and more adequate than others to reach the optimal solution in a reasonable computational time. This is particularly true in the noiseless simulation, in which case the good quality of the solutions reached by the Cobyla optimizer is not dependent on the ansatz. However, for quite all optimizers, and also for Cobyla optimizer in the presence of errors, the quality of the final solution depends on the ansatz.

As far as the ansatzes are compared, they present both different convergence rates and different final $E_{min}$ reached. Moreover, some show delayed convergent behavior, thus suggesting that their inherent structure affects the training process. In particular, the PauliTwo ansatz soon reaches a good quality solution. After PauliTwo, the fastest ansatzes that reach the same solution quality, in the noiseless case, are RealAmplitude with either full or pairwise entanglement. The linear entanglement construction is less powerful as a parametrization, but it converges fast toward the optimal solution. Instead, circular entanglement on both TwoLocal and RealAmplitude structures is associated with a lower convergence rate. On the other hand, the full entanglement ansatzes converge in most cases to higher final energies, i.e., worse solutions, with noticeable effects in noisy simulations. With regard to the Cobyla optimizer, all the ansatzes converge to similar solutions in the noiseless case. In the presence of noise, good solutions are obtained soon with PauliTwo ansatz, and the best final solutions are obtained by both TwoLocal and RealAmplitude ansatzes with both linear and pairwise entanglement. Therefore, PauliTwo ansatz should be chosen to obtain a solution after very few epochs, while one of the latter ones could be preferred if a slightly longer computational time is acceptable.

Comparison of Figs. 2 and 3 clearly show the effect of noise and errors in the computation. Some ansatzes allow to approach the same minimum energy as in the noiseless case but require more epochs to converge. For other ansatzes, the solution converges to values appreciably different between the noisy and noiseless cases. Compared to the noiseless situation, the optimizers drift from the full convergence due to the effect of noise. However, for all ansatzes, target values are obtained after a few epochs by the Cobyla optimizer, even in the noisy case. Therefore, the convergence rate is influenced by errors. Still, stable solutions are found by the Cobyla optimizer immediately with the PauliTwo ansatz, designed to avoid plateau during training and after a few epochs with the others. This comparison thus reveals that the effect of quantum and measurement noise is quite relevant: noise hinders the convergence of the VQE in almost every situation by either slowing down the convergence rate or shifting up the value of the minimum energy, i.e., the quality of the solution found. However, the effect of the error is neglectable if the Cobyla optimizer and the appropriate ansatz are chosen.

**Figure 4:**
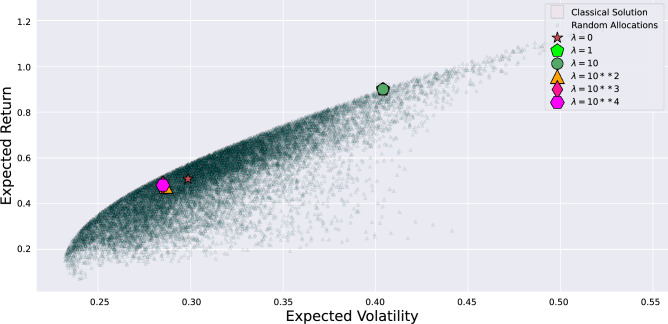
Effect of variable penalty coefficient, used to transform the constrained into an unconstrained problem. Dots represent the random sampling of possible solutions satisfying the constraint of the continuous problem. Among them, a few are also possible solutions to the integer PO problem. The square corresponds to the optimal solution found by the classical branch-and-bound method. The other symbols correspond to the optimal solutions found to the QUBO problem, with constraints embedded in the objective function by setting different values of the penalty coefficient. For each penalty value, the evaluation of the best PO has been performed taking the average of 2000 runs from the quantum circuit optimized via the VQE.

Secondly, the effect of the parameter $\lambda$, used as penalty coefficient to weight the constraint satisfaction with respect to the objectives, to transform the constrained (12) into unconstrained quadratic problem (13), is investigated. Fig. 4 reports the results obtained.

In detail, Fig. 4 reports the expected return vs. volatility of different portfolios. Dots represent the random sampling of possible solutions satisfying the constraint of the continuous problem (6). The set of these points evidences the Markowitz efficient frontier, which is the set of solutions with maximal return for each volatility, where the optimal solution should lie. Among them, a few are possible solutions to the integer PO problem (7). The square corresponds to the optimal solution found by the classical branch-and-bound method. The other symbols correspond to the optimal solutions to the integer (7) or binary problem (12), with constraints embedded in the objective function (13) by setting values of the penalty coefficient $\lambda$ of different orders of magnitude. The optimal solutions are obtained after 250 epochs on the simulated IBM Cairo machine using the Cobyla optimizer and the TwoLocal linear entanglement ansatz.

From Fig. 4, it can be noticed that all optimal solutions lie close to the Markovitz frontier. Moreover, while the solution corresponding to $\lambda =0$ does not necessarily respect the budget constraints, as long as moderated $\lambda$ values are used, the optimal solutions overlap with the classical result. Instead, over this optimal region, as $\lambda$ is still increased by powers of 10, sub-optimal solutions are obtained since the penalty term in (16) becomes dominant respect to the coupling terms and thus hinders the mapping onto the original problem. The quadratic term proportional to lambda, if not properly balanced, modifies the spectral properties of the Hamiltonian, shifting the energies and the eigenstate of the unperturbed problem (i.e., the Hamiltonian of the unconstrained problem). For these experiments, the interval $1 \le \lambda \le 10$ guarantees the convergence of the solution to the classical findings. In general, these results allow individuating the optimal value of $\lambda$ within the same order of magnitude of the fraction between the objectives and the constraint satisfaction quadratic term.

These results confirm that the correct choice of the penalty coefficient $\lambda$ is very important, and for the PO problem, the workable values are found in these experiments. In general, constraints can be divided into *hard* and *soft*^39^. A hard constraint must be satisfied, then $\lambda$ must be large enough to preclude violations. Instead, in this case, a soft constraint can be used, according to PO practical applications, so slight violations can be tolerated, and a moderate penalty value is sufficient. Experiments show that a too-large penalty value can negatively influence the solution process since the penalty terms overwhelm the original objective function information, which introduces difficulties in distinguishing the quality of different solutions. On the other hand, a too-small penalty value offers solutions not adequately in accord with the budget. The *Goldilocks region*^39^, the interval of values that work for the PO problem, is found above.

### Experiments on real quantum computers

In this section, experiments are run on real NISQ devices, detailed in the Methods Section. A fixed number of 200 epochs was chosen. The hyperparameters present the best behavior in the simulated runs, i.e., the QUBO model is obtained by $\lambda =10$, and the VQE algorithm employs the Coybla classical optimzer and the TwoLocal linear entanglement ansatz.

**Figure 5:**
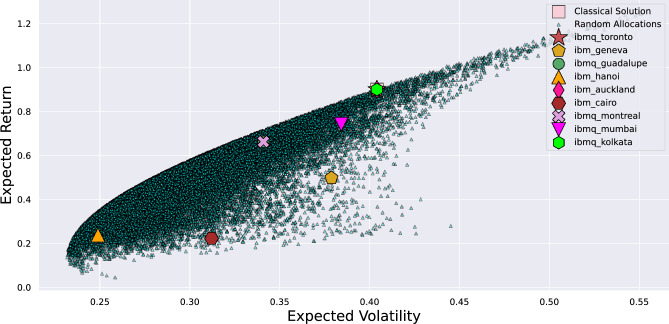
Results of experiments run on different real quantum devices with $\lambda =10$. Dots represent the random sampling of possible solutions satisfying the constraint of the continuous problem. The square corresponds to the optimal solution found by the classical branch-and-bound method. The other symbols correspond to the optimal solutions found to the QUBO problem, by means of different IBM quantum computers. For each quantum hardware, the evaluation of the best PO has been performed taking the average of 2000 runs from the quantum circuit optimized via the VQE.

The experimental results are shown in Fig. 5. In particular, the figure represents the return and volatility of the solution. The dots represent the random sampling of possible solutions to the continuous problem. Among them, a few are also possible solutions to the integer PO problem. The classical solution of the integer problem, which lies on the Markowitz efficient frontier, is indicated by a square. The other symbols indicate optimal solutions found by employing different IBM quantum computers.

From Fig. 5, it can be noticed that the solution found by some real quantum computers is perfectly matching with the classical solution. In particular, among those detailed in the Methods section and tested here, the following are those with an optimal solution: Toronto, Kolkata, and Auckland.

**Figure 6:**
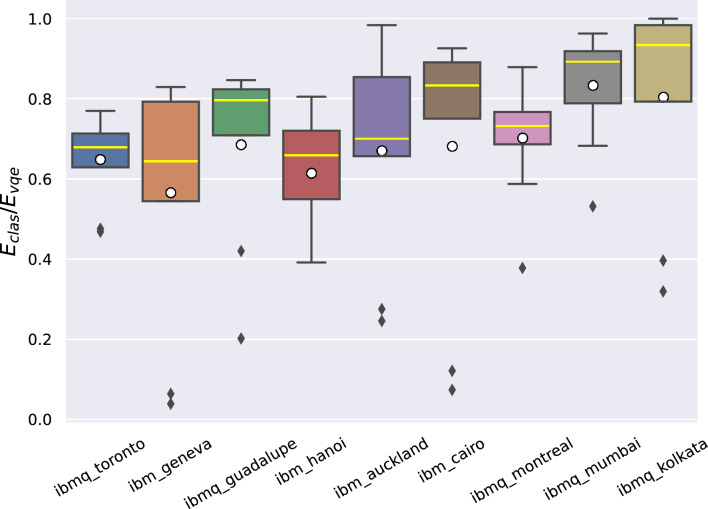
Results of experiments run with the nine ansatz considered, on different real quantum devices, ordered according to growing quantum volumes. The box extends from the quartile $Q_{1}$ to $Q_{3}$ of the data, with a yellow line at the median ($Q_{2}$). The black lines extend from the edges of the box to show the range of the data. Here a standard approach is followed, as they extend to a maximum of $1.5 * (Q3 - Q1)$ from the edges of the box, ending at the farthest data point in the interval. Data outliers are plotted as black squares, while the white dots represent the mean of the result distribution for each hardware.

A more detailed discussion can be done on the basis of Fig. 6, which presents the fraction between the minimum energy found by the classical method and by VQE run on real devices. In particular, optimal results should approach 1, and the figure shows the statistics of the results over repetitions of the simulations on each real device, performed with different ansatzes. In particular, the devices are shown in order of growing quantum volume.

From Fig. 6, it can be noticed that there is an increasing trend both in the mean and in the median, as the QV of the quantum computer grows.

These results show that both the mapping of the ansatz structure on the hardware topology and the quantum volume is of pivotal importance for reaching the desired convergence. The topology of a quantum computer refers to the physical arrangement of qubits: while ansatzes connecting only the nearest qubits can be mapped efficiently, those entailing long-range connections require an overhead of gates that ultimately increases the depth of the circuit and hence foster an increase of the overall error rate during computation. On the contrary, densely structured ansatzes, like the TwoLocall full entanglement, provide a robust and potentially more expressive benchmark to explore the parameter space, and thus to find the global minimum of the objective function. In this sense, a balance needs to be found between the expressiveness of the ansatz and the mapping on the hardware topology. Ultimately, a higher QV allows to perform computation on a deeper circuit without an exponential increase of the error rate: hence, as 6 suggests, higher quantum volumes, as for *ibm kolkata*, allows to run efficiently largely parametrized ansatzes, which converge better to the global optimum of the problem.

## Conclusions and future perspectives

In this paper, the Portfolio Optimization problem was approached by Quantum Computing, in particular by translating the quite general quadratic problem formulation into a Quadratic Unconstrained Binary Optimization, mapped to a Hamiltonian, whose minimum eigenvalue is approximated by the Variational Quantum Eigensolver, and corresponds to the optimal portfolio.

In particular, different hyperparameters of this approach are analyzed, i.e., the penalty coefficient that enables the transformation of the problem from constrained to unconstrained, the type of parametric wave-function (ansatz), and the optimizer employed in VQE. Moreover, experiments were run on both simulators and on different real quantum computers.

The importance of selecting a proper ansatz and optimizer for the VQE and a proper penalty coefficient was revealed. Moreover, the best choices were individuated, in order to solve the most efficient PO by VQE, even in presence of quantum hardware noise. Furthermore, the relation between the quality of the solutions found by VQE and the characteristics of the quantum computers was found to be dependent on the intrinsic properties of quantum processors. Even though this is well known in the literature, here it has been proven and validated experimentally.

Finally, solutions found were bench-marked with the classical solution. Albeit the scale of the system considered is not matching with realistic requirements, the solutions of the VQE on NISQ devices reveal promising features, both in terms of complexity and the solution quality. In summary, the present study suggests as best practice to follow when dealing with PO on QC, particularly when the VQE is used: a gradient-free optimizer such as *Cobyla*, an ansatz with linear or pairwise entanglement structure, i.e. expressive and yet not too complex when transpiled on the quantum hardware, and a quantum computer with large quantum volume.

Future perspectives consist in solving real-life portfolio optimization problems, with higher market size, as soon as quantum devices with appropriate characteristics will be available. Moreover, future work includes the formalization of the problem in the most advanced ways available. Finally, the effect of the topology of the hardware on quantum variational algorithms will be matter of a follow-up investigation.

### Supplementary Information

Supplementary Information 1.

Supplementary Information 2.

## Data Availability

All data analysed during this study are included in this published article and its supplementary information files.
