# Supplementary material for: Best practices for portfolio optimization by quantum computing, experimented on real quantum devices
Source: Sci Rep. 2023 Nov 8;13:19434. doi: 10.1038/s41598-023-45392-w (PMC10632408; doi:10.1038/s41598-023-45392-w)
Supplement: Supplementary file 2 — Supplementary Information 2. [file 41598_2023_45392_MOESM2_ESM.pdf]

Supplemental Experiments: Best practices for portfolio optimization by quantum computing, experimented on real quantum devices

Giuseppe Buonaiuto[1], Francesco Gargiulo[1], Giuseppe De Pietro[1], Massimo Esposito[1], and Marco Pota[1]

National Research Council of Italy (CNR), Institute for High Performance Computing and Networking (ICAR), Naples, 80131, Italy
marco.pota@icar.cnr.it

1 Experiments

Here, the convergence of experiments performed by using different ansatzes and optimizers are reported, in addition to those presented in the main paper. Both figures show the convergence rate, during epochs, towards the minimum energy E_{min}, for two different set of data, extracted by *yahoofinance*. Notice that the first dataset is made of three assets (Supplementary Fig. 1), while the second of four assets (Supplementary Fig. 2): a variable number of asset allows to draw robust conclusion about the performances the optimizers and of the ansatz, regardless of the number of assets considered. In particular, the experiments are performed with the QASM quantum simulator, importing the noise structure of *IBM Cairo*, hence simulating a real quantum device. In both cases, the experiments are performed over a set of nine ansatzes and three possible optimizers. Regarding the comparisons among classical optimizers, both figures allow to draw the same considerations expressed in the main work. First, both the Cobyla and the NFT optimizers foster a rapid convergence towards low values of the energy for every ansatz, while SPSA presents a delayed behavior. On the other hand, the NFT optimizer, contrarily to the others, experiences relatively unstable behavior, with highly oscillating trajectories for each ansatz. Moreover, all optimizers are relatively robust against statistical noise, but a more oscillating behavior is obtained in noisy simulations using SPSA or NFT optimizers. As far as the ansatzes are compared, they present both different convergence rates and different final E_{min} reached. In the presence of noise, good solutions are obtained soon with PauliTwo ansatz, and the best final solutions are obtained by both TwoLocal and RealAmplitude ansatzes with both linear and pairwise entanglement, hence in line with the experiment presented in the main text.

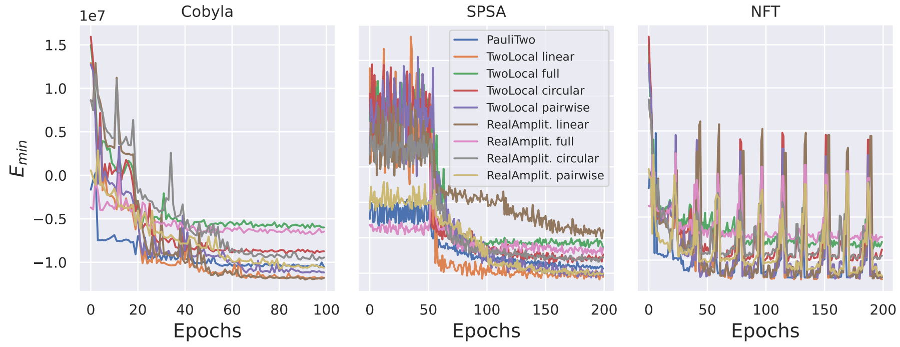

Supplementary Fig. 1. Noisy experiments, performed on IBM QASM simulator, by importing IBM Cairo quantum computer noise model. Convergence of the solutions towards the optimal one during training epochs, evaluated with different optimizers and ansatzes. Here data are taken from yahoo finance, for three assets, namely ACM, AQN, NXP, within a temporal range between 2011/12/23 and 2022/10/21.

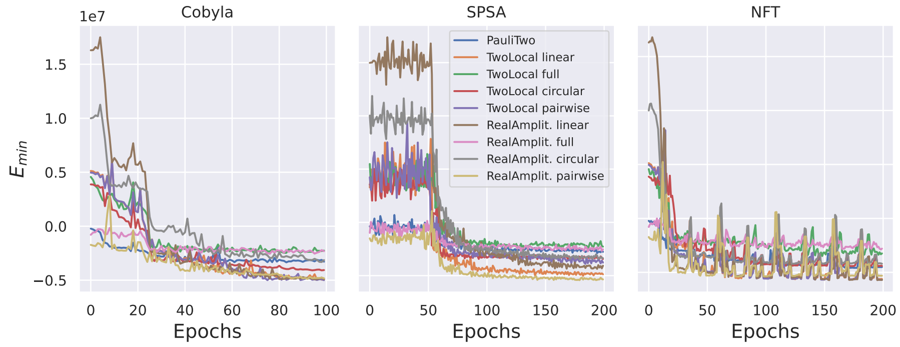

Supplementary Fig. 2. Noisy experiments, performed on IBM QASM simulator, by importing IBM Cairo quantum computer noise model. Convergence of the solutions towards the optimal one during training epochs, evaluated with different optimizers and ansatzes. Here data are taken from yahoo finance, for four assets, namely SPXX, RZB, SLCA, EGY, within a temporal range between 2011/12/23 and 2022/10/21.
